# Epigenetics: New Insights into Mammary Gland Biology

**DOI:** 10.3390/genes12020231

**Published:** 2021-02-05

**Authors:** Elitsa Ivanova, Sandrine Le Guillou, Cathy Hue-Beauvais, Fabienne Le Provost

**Affiliations:** GABI, INRAE, AgroParisTech, Université Paris-Saclay, 78350 Jouy-en-Josas, France; elitsa.ivanova@inrae.fr (E.I.); sandrine.le-guillou@inrae.fr (S.L.G.); cathy.hue-beauvais@inrae.fr (C.H.-B.)

**Keywords:** mammary gland, epigenetic regulations, lactation, DNA methylation, non-coding RNA

## Abstract

The mammary gland undergoes important anatomical and physiological changes from embryogenesis through puberty, pregnancy, lactation and involution. These steps are under the control of a complex network of molecular factors, in which epigenetic mechanisms play a role that is increasingly well described. Recently, studies investigating epigenetic modifications and their impacts on gene expression in the mammary gland have been performed at different physiological stages and in different mammary cell types. This has led to the establishment of a role for epigenetic marks in milk component biosynthesis. This review aims to summarize the available knowledge regarding the involvement of the four main molecular mechanisms in epigenetics: DNA methylation, histone modifications, polycomb protein activity and non-coding RNA functions.

## 1. Introduction

The mammary gland is a complex organ that undergoes important modifications during its development and at each cycle of reproduction. The precise and complex regulation of mammary development has been extensively studied over the years at the genetic, physiological and morphological levels. Recent reports have assessed the potential implications of epigenetic control of normal development and regulation of particular cell types in the mammary gland during the different mammary gland stages.

In this paper, we review the available data on four main molecular mechanisms involved in epigenetics (DNA methylation, histone modifications, polycomb protein activities and ncRNA functions) in mammary gland biology. Data concerning mammary cancers, as well as epigenetic modifications due to the environment, health and diet, are not included in this review.

## 2. Mammary Gland

**Figure 1:**
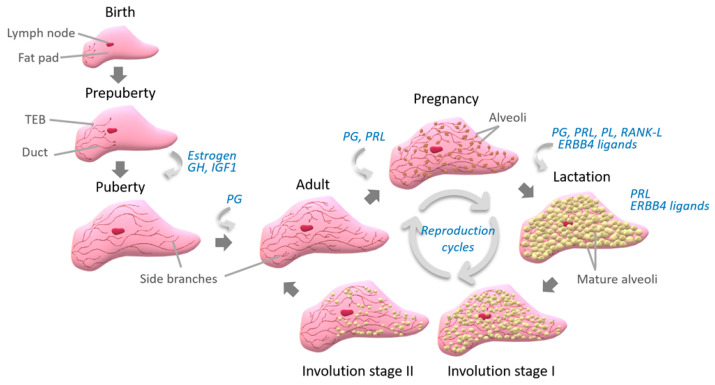
Principal stages of the mammary gland development throughout the lifetime. Major hormones that control development are outlined in italics and blue. TEB: terminal end bud. GH: growth hormone. IGF1: insulin-like growth factor-1. PG: Progesterone. PRL: Prolactin. PL: Placental lactogen. ERBB4: Erb-B2 Receptor Tyrosine Kinase 4. RANK-L: receptor activator of nuclear factor kappa-B ligand.

The mammary gland is a complex organ containing two compartments, the epithelium and the stroma (for review, see [1]). Ducts and milk-producing alveolar structures constitute the epithelium. The majority of epithelial cells are secretory cells. These cells are subject to functional differentiation during pregnancy in order to produce milk later in lactation. Myoepithelial cells surround epithelial cells and contract, allowing the delivery of milk. The ducts and alveoli are embedded in the stroma, a connective tissue. This tissue is composed mainly of adipose tissue and contains blood vessels, fibroblasts, neurons, and haematopoietic cells. The development of the mammary gland occurs throughout the lifetime, and the lactating mammary gland is the result of the succession of distinct hormone-regulated stages (Figure 1). The mammary anlage is established during foetal development. Then ductal elongation and branching take place principally after puberty. During pregnancy, alveolar proliferation occurs; however, functional differentiation is not achieved until parturition and during lactation. Involution is initiated by the loss of suckling stimuli and the pressure build-up due to cessation of milk removal. During this stage, massive cell death, collapse of the alveoli and remodelling of the epithelial compartment to restore a simple ductal structure are observed. The process reinitiates for subsequent pregnancies. The regenerative capacity of the mammary gland is enabled by mammary stem cells [2].

## 3. Epigenetic Modifications

**Figure 2:**
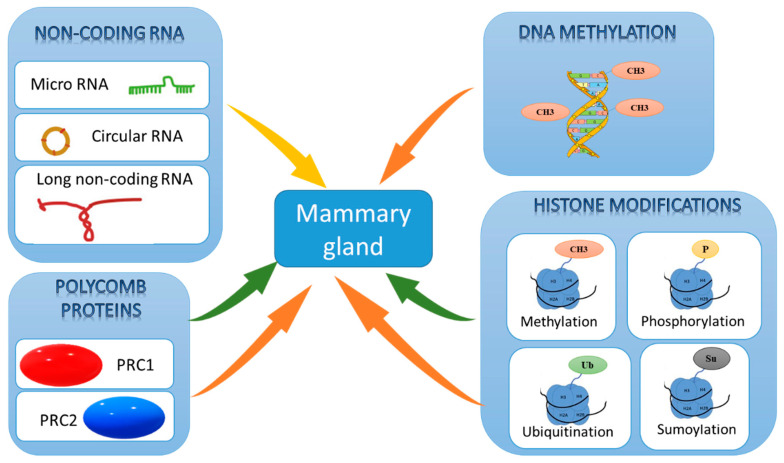
Main epigenetic modifications impacting mammary gland development. Post translational regulation (yellow arrow), alteration of chromatin structure (green arrow), and gene activation or inactivation (orange arrow).

The concept of epigenetics describes mitotically stable states and changes in gene activity that do not involve modifications of the DNA sequence, thus providing a supplementary layer of information and control [3]. Epigenetic mechanisms correspond to post-translational modifications of histones and covalent chemical modifications of nucleic bases that define chromatin structure (Figure 2 and Table 1) [4,5]. Through these epigenetic mechanisms, cells integrate environmental stimuli to coordinate a wide range of DNA processes, including gene transcription.

Epigenetic modifications are not restricted to a specific life stage of an organism but continue throughout the lifespan [6]. Nevertheless, they more commonly arise during stages of development and cell proliferation [7]. As discussed previously, the mammary gland goes through several developmental periods including prenatal, postnatal, and puberty. During this time, the mammary gland might be more sensitive to epigenetic modifications and disruptions [8,9].

### 3.1. DNA Methylation

DNA methylation is a process in which a methyl group is added to the carbon-5 position of a cytosine with temporal and spatial precision [10]. This mechanism, mediated by DNA methyltransferases (DNMTs) and DNA demethylases, is important in CpG islands, regions where a cytosine nucleotide is followed by a guanine nucleotide in the sequence of bases [11]. These CpG islands are enriched at promoters, and the associated genes can be silenced upon CpG methylation [12]. Inversely, gene body methylation correlates with transcriptional activation [13]. Three different DNMTs (DNMT1, DNMT3A, and DNMT3B) catalyse DNA methylation and can behave both as transcription enhancers and inhibitors [14,15]. CpG islands are demethylated by ten-eleven translocation (TET), a chromatin modifier that allows the conversion of 5-methylcytosine (5-mC) into 5-hydroxymethylcytosine (5-hmC) to activate DNA demethylation. In this way, TET family proteins (TET1, TET2, and TET3) regulate embryonic and adult stem cell homeostasis [14,16]. DNA methylation can also be found at non-CpG sites, this is referred to as non-CpG methylation. While it was first discovered in the plant genome [17], it has also been found in embryonic stem cells and brain tissue [13,18]. DNMT3A and DNMT3B are known to catalyse non-CpG methylation [19,20].

### 3.2. Histone Modifications

Covalent histone modifications, such as acetylation, methylation, phosphorylation, ubiquitination and sumoylation, are dynamic and can regulate gene expression [21,22]. The crosstalk between these marks and DNA methylation plays a key role in the epigenetic regulation of genome expression [23]. Histone modifications may activate or inactivate neighbouring genes, alter chromatin structure and conformation or recruit transcriptional activators/suppressors [24,25,26,27]. At first believed to be an irreversible process, histone methylation was thought to only be removed by histone eviction or dilution through DNA replication. However, after the discovery of enzymes that can demethylate specific Lys residues on histones and, in some cases non-histone substrates, this model changed. Histone methylation and demethylation are very important for developmental control, cell fate decisions, and disease [28].

Histone methylation can designate both transcriptionally active (H3K4me3) and inactive (H3K27me3) regions [29]. Bivalency refers to the presence of both H3K4me3 and H3K27me3 at promoters [30]. Genes with bivalent domains are primed for differential expression upon differentiation; they are often found in embryonic stem cells but have been discovered in adult stem cells as well. Studies suggest that the resolution of a bivalent domain is required to regulate development and commit to lineage choice [31]. The chromatin state also influences the histone modification rate. Chromatin can be untranscribed and compact, defined as heterochromatin, or can be transcribed and loose, defined as euchromatin [32]. Heterochromatin plays a role in transcription regulation by limiting access to DNA and impacting the location of nucleosomes. Histone modifications are catalysed by specific enzymes, such as Histone acetyltransferase (HAT), Histone methyltransferase (HMT), Protein arginine methyltransferase (PRMT), Histone deacetylase (HDAC) and Lysine demethylase (KDM) [33].

### 3.3. Polycomb Proteins

Epigenetic regulation can be mediated by polycomb-group proteins (PcGs), which are transcriptional regulators that play a role in establishing and maintaining epigenetic memory during development. In mammals, PcGs form two complexes, Polycomb Repressive Complexes 1 and 2 (PRC1 and PRC2, respectively). PRC2 is the main mammalian complex responsible for H3K27 trimethylation and is integral to chromatin organization. Important targets of H3K27 methylation include genes involved in development, stem cell maintenance, and differentiation [34]. PRC2 is comprised of several subunits, which include either Enhancer of Zeste Homologue 2 (EZH2) or EZH1 [35] in combination with Suppressor of Zeste 12 protein homologue (Suz12) and Embryonic Ectoderm Development (EED) [36].

### 3.4. Non-Coding RNAs

Non-coding RNAs (ncRNAs) are genes that are transcribed but not translated into proteins and play an important role in epigenetic regulation [37,38,39]. There are several classes of ncRNAs due to their functions as housekeeping or regulatory RNAs. Several long non-coding RNAs (lncRNAs), microRNAs (miRNAs), and circular RNAs (circRNAs) are involved in epigenetic regulation. Depending on their function, lncRNAs, which are transcripts of greater length than 200 nucleotides, can be classified as signals and decoys. In this case, they are associated with gene activation and suppression. They can also be classified as guides that regulate gene expression by recruiting enzymes that modify chromatin. They can be defined as scaffolds that allow the formation of ribonucleoprotein complexes by recruiting proteins [40]. However, these functions are not exclusive; that is, multiple functions can be performed by one lncRNA. miRNAs are small, conserved ncRNAs (18–22 nt in length) [41] that participate in post-transcriptional gene expression through negative regulation of translation or through mRNA degradation. circRNAs are ncRNAs generated from back-splicing reactions of linear RNAs. The most important function of circRNAs is to act as sponges for miRNAs in cells by increasing the number of available miRNA binding partners.

Many studies have been conducted in the context of breast cancer and tumorigenesis; however, epigenetic control is crucial in normal mammary biology, as shown in several recent studies [42,43]. The mammary gland has the capability to undergo cycles of cell proliferation, differentiation and apoptosis during adult female life. Epigenetic regulation plays crucial roles in these different processes. In this review, we propose an overview of epigenetics in normal mammary gland development and lactation.

## 4. DNA Methylation in the Mammary Gland

**Figure 3:**
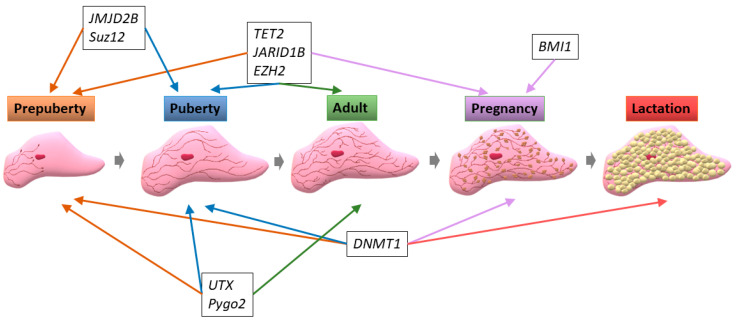
Epigenetic modulating proteins with a role in mammary gland development: Mammary gland development occurs throughout the lifetime and can be divided into several distinct stages some of which are represented here. Multiple proteins, involved in epigenetic regulation and mentioned in the text boxes above, play a role in this development largely through influencing mammary stem cell quiescence and activation, as well as luminal differentiation and lineage commitment. Colour-coded arrows show which stages these proteins modulate.

DNA methylation modulating proteins are involved in the regulation of different mammary gland development stages (Figure 3).

DNMTs mediate DNA methylation and their role in virgin and pregnant mice has been investigated using the DNMT inhibitor azacitidine (AzaC). A reduction in the number and size of ductal and alveolar structures after AzaC treatment is found regardless of pregnancy status and is likely caused by decreased cell proliferation of mammary epithelial cells [44]. The DNA methylation patterns are relatively similar; however, genes with low expression are preferentially hypermethylated due to age, not pregnancy [44]. Interestingly, similar DNA methylation patterns are found between pregnant and retired breeder mice [44]. Pregnancy is found to induce substantial and persistent changes to DNA methylation of sites that bind Signal Transducer and Activator of Transcription (STAT) 5A and genes upregulated during pregnancy [43]. DNMT1 is necessary for ductal and terminal end bud (TEB) development. In fact, deletion of DNMT1 in mice leads to a significant reduction in mammary stem/progenitor cells [45].

Another DNA methylation modulating protein is TET2. It is the most highly expressed TET family protein in mammary tissue (The Human Protein Atlas). A mouse model with mammary-specific TET2 deletion presents with impaired luminal lineage commitment resulting in enhanced ductal branching and an increased number and size of TEBs, as well as fibrosis and hyperplasic lesions in virgin heterozygous and knockout mice compared to wild type mice [46]. Lactating heterozygous and knockout mice are found to have defective luminal-alveolar development, with fewer lobulo-alveoli; those that are formed are deficient in lipid droplet-like morphology, resulting in reduced milk production. These findings suggest that TET2 plays an important role in directing the differentiation of mammary stem/progenitor cells [46]. Moreover, TET2 forms a chromatin complex with the transcription factor Forkhead box P1 (FOXP1), which then regulates the demethylation of *Estrogen receptor 1* (*Esr1*), *GATA binding protein 3* (*Gata3*), and *Forkhead box A1* (*Foxa1*). These three genes are known to be involved in mammary luminal lineage specification [47,48].

Lactation performance (milk quantity and milk composition) is under the control of several important pathways, such as the hypothalamic-pituitary-adrenal (HPA) axis pathway, which regulates the expression of Growth Hormone (GH) and Prolactin (PRL). Paired-like homeodomain 1 (PITX1), located upstream of the HPA axis pathway, has been shown to activate the *Pro-opiomelanocortin* (*Pomc*) gene and to interact with Pituitary-specific positive transcription factor 1 (Pit1), resulting in differential expression of GH, PRL, and Thyroid-Stimulating Hormone (TSH), which in turn affects lactation performance in mammals [49]. In goats, the overall DNA methylation status of a *PITX1* CpG island in the mammary gland showed hypermethylation in tissue from the dry period (a period of rest between lactations) and hypomethylation in tissue from the lactation period [50].

In the same study, several important transcription factors, CCCTC-binding factor (CTCF), STAT, SMAD, CDE promoter element (CDEF), Specificity Protein 1 (SP1), Kruppel-Like Factors (KLFs), and zinc finger transcription factors, were predicted to bind to the same *PITX1* CpG island. These transcription factors are known to influence various biological functions that could affect *PITX1* and therefore lactation performance. CTCF binds the gene *Imprinting control region* (*ICR*), thereby regulating gene expression [51]. Proteins in the STAT family represent some of the main components of the JAK/STAT pathway, which plays a role in the development and function of mammary epithelial tissue [52]. SMAD signalling influences mammary gland differentiation via the JAK/STAT pathway [53]. CDEF could regulate the epithelial cell cycle [54]. SP1 and KLFs could play a role in the expression of genes with GC-rich promoters [55].

In the lactating bovine mammary gland, a 10 kb region upstream of the *αS1-casein* (*Csn1S1*) gene is hypomethylated. Three CpG dinucleotides are methylated following *E. coli* or *S. uberis* infection, which is associated with chromatin condensation, resulting in the cessation of *αS1-casein* expression [56]. These three CpG dinucleotides are also methylated in healthy mammary glands following an 8-day non-milking period [57] or during pregnancy [58].

DNMT1 is expressed at a higher rate in cloned lactating cows than in non-cloned lactating cows. This is linked to a higher apoptotic rate and lower rate of *αS2-casein* transcription [59].

Mammary gland fat development is influenced by multiple genes containing CpGs that are differentially methylated before, during, and after pregnancy in humans, for example, *SH3 and PX domains 2B* (*sh3pxd2b*), *RAR-related orphan receptor C* (*rorc*), and *AT-rich interaction domain 5B* (*arid5b*) [60].

Transcription factor E74-like factor 5 (Elf5) controls the differentiation of mammary luminal progenitor cells into alveolar cells. Methylation of the *Elf5* promoter maintains the stem cell and myoepithelial lineages, whereas loss of this methylation is a mark of the luminal lineage. *Elf5* promoter methylation is therefore lineage-specific [61]. Similarly, transcription factors associated with luminal differentiation (e.g., GATA3) are found to be gene body methylated (activated) in human mammary luminal cells [62].

**Figure 4:**
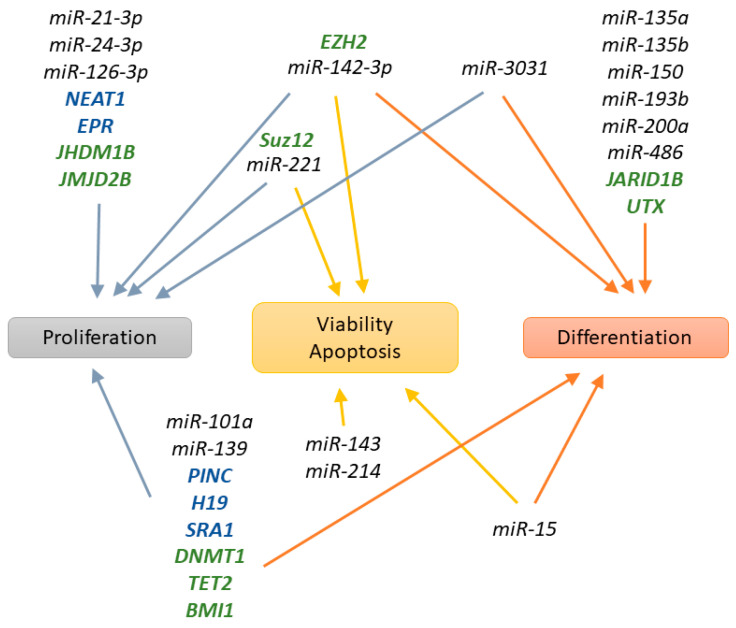
Cell processes modulated by epigenetic regulators: The epigenetic modulating proteins (in green), microRNAs (in black), and long non-coding RNAs (in blue) discussed in this review can be categorized based on which cell process they play a role in. Colour-coded arrows show which processes the mentioned epigenetic regulators are involved in.

DNA methylation modulating proteins affect the expression of multiple genes that play a role in mammary gland development. As a result, DNA methylation plays an indirect role in the formation of TEBs, ductal elongation, lobulo-alveolar, and fat development. It affects lactation performance and marks mammary epithelial cells with a different methylation profile depending on the mammary gland stage of development, whether it is a question of age or reproduction cycle. On a cellular level, DNA methylation is important for the regulation of mammary epithelial cell proliferation, viability, and differentiation (Figure 4). It is indisputable that DNA methylation is at least partially responsible for mammary gland development regulation; however, the intricate relationships between the different genes it affects have yet to be completely understood.

## 5. Histone Modifications in the Mammary Gland

In this review, we will discuss modifications on histone 3 (H3) and their effects on mammary gland development. Mouse mammary stem/basal, luminal progenitor cell, and mature luminal cell subsets display distinct patterns of H3K27me3 in the steady state. The mammary stem/basal cell subset is found to have the lowest H3K27me3 levels; higher levels of H3K27me3 coincide with reduced gene expression, and H3K27me3 levels increase upon cell specification. This suggests that mammary epithelial cell differentiation is dependent on the narrowing of transcriptional programmes and suppression of alternate cell fates [63].

Multiple histone modifying proteins are important for the regulation of mammary gland development during the various stages (Figure 3) and the control of several mammary epithelial cell processes (Figure 4).

Lysine-specific demethylase 5B (JARID1B), also called PLU1 or KDM5B, is an epigenetic regulator. This histone demethylase converts tri- and dimethylated lysine 4 on histone H3 (H3K4me3 and H3K4me2, respectively) to the monomethylated form (H3K4me1) [64]. It is a part of the JARID1 protein family, which contains proteins that possess H3K4 histone demethylase activity in vitro and in vivo [65]. H3K4me3 is a mark of positive transcription; therefore, its demethylases can act as transcriptional repressors that silence gene expression [66,67]. Nevertheless, JARID1B can activate gene expression when predominantly recruited to intragenic regions according to Xie et al. [68]. Surprisingly, a more recent study from Kidder et al. contradicted this JARID1B recruitment site in embryonic stem cells. While it did suggest that JARID1B removed intragenic H3K4me3, it also showed that it was predominantly bound to active promoters and enhancers, in correlation with H3K4me3 marks [69]. The latter is also in line with the findings of Ram et al., further contradicting Xie et al. [70]. It is difficult to determine the reason for these different results, especially since both Xie et al. and Kidder et al. agree that JARID1B depletes intragenic H3K4me3. Further studies are required to elucidate the role of JARID1B in gene expression.

JARID1B is expressed in the mammary gland and several components of the female reproductive system. Female mice lacking JARID1B exhibit delayed mammary gland development, reduced fertility rate, lower estrogen levels in circulating blood, and an altered transcription programme in the mammary epithelium [71]. The loss of JARID1B leads to a decrease, rather than an increase, in H3K4me3 levels. In fact, in a mouse model, JARID1B has been shown to act as a transcriptional activator by recruiting the luminal transcription factor GATA3 to its target genes, such as *Foxa1* and *Stat5a* [71].

FOXA1, a transcription factor that plays an important role in modulating estrogen receptor α (ERα) binding to many of its target genes, is indispensable in long-range chromatin remodelling, thereby allowing ERα to access its targets [72]. In the mammary gland, FOXA1 is also essential for ductal invasion during puberty but is not required for alveologenesis during pregnancy and lactation [73]. *FoxA1* gene body methylation level is higher in mammary tissue from early parous women (full-term pregnancy under the age of 25) than in mammary tissue from nulliparous women [74]. *FOXA1* is downregulated in JARID1B-lacking mice, suggesting that an epigenetic regulator can directly affect another factor with chromatin remodelling functions.

Lysine-specific demethylase 6A (UTX), also named KDM6A, demethylates H3K27me2/3 and is, therefore, a transcriptional activator [75]. In a mammary luminal cell line, the depletion of UTX leads to the loss of luminal transcription factors, leaving behind cells with basal characteristics [76]. An in silico correlation between GATA3 and UTX was validated in vitro, and the complex formed was found to activate multiple genes, including *Dicer* and *UTX* itself. Dicer plays a key role in the biogenesis of small regulatory RNAs, such as miRNAs and siRNAs [77].

Another demethylase that plays a role in mammary gland epigenetic regulation is JmjC-domain-containing histone demethylase 1B (JHDM1B), also known as FBXL10. This protein demethylates H3K4me3 and H3K36me2, thus removing active transcription marks and inhibiting gene expression [78]. Galbiati et al. reported that JHDM1B knockdown in two mammary epithelial cell lines leads to an increase in the levels of either H3K4me3 or H3K36me2, depending on the cell line. This suggests that JHDM1B is involved in the demethylation of these residues. Knockdown of JHDM1B leads to increased cellular proliferation in one cell line and increases colony-forming ability in both cell lines as well as greater invasiveness and staminal potential, the latter of which explains the ability of knockdown cells to form mammospheres [79]. This suggests a role for JHDM1B as a tumor suppressor with control over cell cycle progression in mammary epithelial cells. These results are consistent with the findings of Penzo et al. [80]; however, they differ from Kottakis et al., where silencing of JHDM1B in the same cell lines through a stable lentiviral system led to cell death [81]. One explanation for these contrasting results could be that Kottakis et al. used a non-inducible experimental approach and achieved a better gene silencing efficiency compared to Galbiati et al. and Penzo et al.

Lysine-specific demethylase 4B (JMJD2B) is a histone demethylase also known as KDM4B. JMJD2B depletion reduces ERα enrichment, and estrogen stimulation leads to decreased H3K9me3 methylation levels at ERα target sites in JMJD2B-depleted T-47D human breast cancer cell line, suggesting that JMJD2B is a regulator of H3K9me3 demethylation [82]. A mouse model of JMJD2B depletion in the mammary gland is found to have delayed ductal morphogenesis and thus mammary gland development [82].

These findings describe the role of H3 modifications in mammary cell fate determination, lineage commitment, and cell cycle progression. Histone modifications are involved in ductal morphogenesis and invasion of the fat pad. Moreover, histone modification regulators have been shown to interact with other epigenetic regulators such as *Dicer* and *FOXA1*. This suggests mammary gland development is dependent on a complex regulatory network involving multiple epigenetic mechanisms.

## 6. Polycomb Proteins Role in the Mammary Gland

PcGs are regulatory elements that play an important role in mammary gland development throughout the different stages (Figure 3), but also modulate different mammary epithelial cell processes (Figure 4).

Suz12 is an essential element of all PRC2 complexes. In vivo, Suz12 loss is lethal [83]. It is thus necessary to study the effects of its deletion in other models. One such model is Suz12-deleted basal-derived organoids [84]. Suz12 deletion leads to the loss of H3K27me3 and H3K27me2. This results in a complete block of normal mammary gland development as well as severely reduced progenitor activity in 3D organoid cultures. PRC2 function is key for the development of the mammary gland since it represses alternate transcription programmes and maintains chromatin states.

EZH2, a SET domain-containing methyltransferase, is responsible for the formation of a di- or trimethyl mark on lysine 27 of Histone H3 (H3K27). This mark is later recognized and bound by PRC1, resulting in transcriptional repression. The specific overexpression of EZH2 in mouse mammary epithelial cells results in a multilayered, disorganized ductal epithelium as a consequence of mammary epithelial cell expansion. This supports a role for PcGs in mammary gland morphogenesis [85]. Conventional knockout mice do not survive past the embryonic stage [86], which makes understanding the role of EZH2 in postnatal mammary gland development and function more difficult. Alternate models to conventional EZH2 knockout mice have therefore been used. A mouse model lacking EZH2 in mammary stem cells was generated by Yoo et al. [87]. Whole mounts of mammary glands collected at day 13 of pregnancy show augmented mammary alveolar content in mice lacking EZH2 compared to controls. Histological analysis of mammary tissue collected at the same stage reveals signs of precocious differentiation of the alveolar epithelium in mice lacking EZH2 compared to controls, signs such as overt lumina, small lipid droplets, and expression of whey acidic protein (WAP), normally detected in late pregnancy [87]. In this mouse model, EZH1 was found to compensate for the absence of EZH2. The same team also performed transplantation experiments using mammary epithelial cells from mature virgin mice. EZH2 knockout or control cells were injected generating similar ductal outgrowths. This is in contrast to the findings of Pal et al., who discussed a 14-fold decrease in the frequency of mammary repopulating cells when a mammary fat pad reconstitution assay was performed with cells lacking EZH2 [63].

Moreover, in another study by Michalak et al., a transgenic mouse model that allows the inducible knockdown and, therefore, the temporal control of EZH2 expression was used. This model showed that EZH2 knockdown in newborn mice stunts mammary gland development in young virgin mice by delaying terminal end bud (TEB) formation and impairing ductal elongation during puberty. Moreover, EZH2 is necessary for maintaining the luminal cell pool, and its knockdown delays lobulo-alveolar expansion during pregnancy [88]. These results underline the importance of EZH2 in mammary luminal cell specification. The reasons for the contradictory results discussed here are difficult to pinpoint, and further studies are required to better understand the role of EZH2 in mammary gland development.

Interestingly, the effects of PRC2 complex member B lymphoma Mo-MLV insertion region 1 homologue (BMI1) deletion are the inverse of those seen for EZH2 knockdown. BMI1 deletion causes premature lobulo-alveolar differentiation [89], suggesting that EZH2 and BMI1 might have opposing roles during pregnancy-induced differentiation of luminal cells.

The hedgehog signalling Pathway components patched 1 (PTCH1) and glioma-associated oncogenes (Gli) 1 and 2 are highly expressed in human mammary progenitor/stem cells cultured as mammospheres but are downregulated when cells are induced to differentiate. Mammosphere size and mammosphere-initiating cell number are found to increase when hedgehog signalling is activated and decrease when it is inhibited. BMI1, a downstream target of the hedgehog pathway, modulates these effects [90].

Pygopus 2 (Pygo2), a co-activator of Wnt/β-catenin signalling, plays a role in histone modification. It was found to enhance the acetylation of lysine 56 on histone 3 (H3K56Ac) in cultured human mammary epithelial cells. Global levels of H3K56Ac are reduced in the absence of Pygo2, while its presence permits the optimal expression of multiple histone genes [91].

## 7. ncRNAs in the Mammary Gland

### 7.1. miRNAs

#### 7.1.1. Characterization of miRNAs

The first analysis of miRNAs in the mammary gland was performed in a study of 20 human tissues, including mammary tissue, by microarray. This study revealed that each tissue has a specific pattern of expressed miRNAs, called its miRNome [92]. Subsequently, additional miRNAs expressed in the mammary gland have been identified by a cloning strategy [93,94]. The evaluation of miRNA expression variations during the different mammary gland stages (virgin, pregnancy, lactation, weaning) was performed. The first data were obtained in mice through the study of at least six stages (including virgin, pregnancy, lactation and involution) using a microarray approach followed by high-throughput sequencing [95,96,97,98]. These studies have demonstrated that each stage has its own signature miRNome, similar to a tissue signature. Avril-Sassen et al. [96] identified groups of miRNAs with similar relative expression patterns over the course of mammary gland development and proposed profiles based on the expression of 102 miRNAs, thus distinguishing distinct stages of mammary gland development in a similar manner to the mRNA expression signature. Llobet-Navas et al. [97] found that 12 miRNAs were preferentially upregulated during involution.

Other studies have compared miRNA expression during different parts of the cycle. In mice, Heinz et al. [99] observed that the majority of differentially expressed miRNAs declined between late pregnancy and lactation. In ruminants, lactating and non-lactating stages (in cows [100,101,102], goats [103], and buffalo [104]) and pregnancy and lactation periods (in sheep (five stages studied by microarray) [105]) have been compared. These studies allowed to highlight miRNAs related to the different stages.

**Figure 5:**
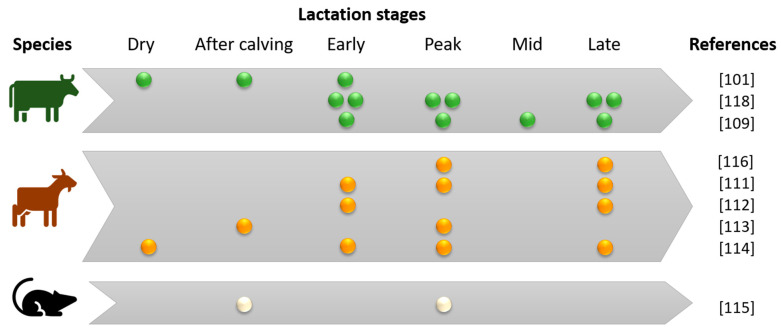
Mammary miRNomes and their variations characterized during lactation stages in cow (green markers), goat (orange markers), and rat species (white markers).

Lactating mammary gland miRNomes were specifically characterized in many species, such as mouse, rat, cow, buffalo, goat, sheep and pig (Table 2). During the lactation stage, the mammary gland undergoes changes, and so does its miRNome. For example, in dairy goats, during early lactation, total number of mammary cells increase for approximately 20% which correspond to mammary growth. The increase in mammary cell number and secretory activity per cell is due to the proliferation and differentiation of mammary secretory cells, and results to the increase in milk production [106,107]. When milk production decreases, particularly during late lactation, the mammary gland undergoes extensive tissue cell apoptosis and remodelling, including changes in cell populations, alveolar structure, and extracellular matrix synthesis. Characterizing miRNomes and their variations during lactation stages improves the knowledge of miRNAs that are crucial for the different biological processes that regulate mammary gland functions. For this reason, several teams have described miRNA expression variations during lactation stages in different species (Figure 5). These data have allowed to describe the highly abundant miRNAs in each species and to identify a lactating mammary gland miRNA signature through comparison across species [101,108,109,110,111,112,113,114,115,116,117,118]. The comparison of miRNA expression in lactating cows, goats and mice and non-lactating humans highlighted 15 miRNAs highly expressed in the mammary gland in all four species (*miR-148a-3p*, *miR-143-3p*, *miR-26a-5p*, *let-7g-5p*, *miR-103-3p*, *let-7f-5p*, *let-7c-5p*, *miR-30a-5p*, *miR-126-3p*, *let-7a-5p*, *miR-378a-3p*, *miR-24-3p*, *miR-200c-3p*, *miR-21-5p* and *let-7b-5p*) (personal data).

With miRNome data, by using bioinformatic tools, target genes to highlight regulatory networks have been obtained [111,112,118]. Ji et al. [112] identified 1487 miRNAs, of which 378 were differentially expressed, by sequencing data analysis of early and late lactation in goat mammary glands. Then, 214 differentially expressed miRNAs and 18 target genes annotated in mammary gland were selected to construct a network comprising 232 nodes (miRNAs and genes) and 335 edges (regulatory relationships between miRNAs and target genes). This analysis revealed miRNAs involved in the same pathways or in different pathways confirming the functional complexity of miRNAs.

In several studies, the transcriptome and miRNome have also been studied in the same samples, and the correlation between mRNA and miRNA expression has been characterized. The analysis of the intersection of the transcriptomic data and putative target genes of differentially expressed miRNAs observed between day 1 and day 7 of lactation in the rat mammary gland allowed to identify 1259 mRNAs overlapping between the two sets of transcripts. The downregulated genes are enriched in pathways involved in lipid biosynthesis [115].

Some authors have studied the expression of specific miRNAs over the different stages, such as *miR-101a* [127], *miR-126-3p* [128], *miR-30b-5p* [129], *miR-424(322)/503* cluster [97], *miR-150* [99], *miR-139* [130], *miR-103* [104] or *miR-142-3p* [131], with the aim of understanding their roles (see § 7.1.2).

Complementary to the study of miRNAs in the whole organ, their expression in the different compartments of the mammary gland, which contain specific cell types, has been characterized. Phua et al. [132] analysed miRNomes of distinct compartments, such as the stroma (essentially composed of adipocytes), mature ducts (constituted by epithelial cells) and TEBs (enriched for stem and progenitor cells) of pubertal mice. They identified a set of specific miRNAs in each compartment, confirming the data obtained by Avril-Sassen et al. [96] showing the specific miRNA expression profiles in stromal *vs* epithelial cells. Moreover, Phua et al. [132] showed that *miR-31* is the most highly enriched miRNA expressed in TEBs. Conversely, *miR-184* expression is significantly enriched in mature ducts compared to TEBs. Interestingly, *miR-184* clusters tightly with a subset of epithelial-specific miRNAs, which includes members of the *miR-183* family (*miR-183*, *miR-96*) and all members of the *miR-200* family.

Bockmeyer et al. [133] performed miRNome analyses on basal and luminal human breast epithelium isolated by laser microdissection. Of the 116 miRNAs detected, eight (*let-7c, miR-125b, miR-126, miR-127-3p, miR-143, miR-145, miR-146-5p* and *miR-199a-3p*) were preferentially expressed in basal cells, and two members of the *miR-200* family (*miR-200c* and *miR-429*) were predominantly expressed in luminal cells.

The global miRNA expression profiles of mouse and human functionally distinct epithelial cell subpopulations (mammary stem cell/basal, luminal progenitor, mature luminal or stromal cells) have been determined, showing unique miRNA signatures characterizing each subset, with a high degree of conservation across the two species [134]. The correlation study between differentially expressed miRNAs and gene expression provides a comprehensive resource for understanding the interplay between miRNA networks and target gene expression, highlighting lineage-specific miRNAs and potential miRNA-mRNA networks. miRNA profiles have also been studied in the COMMA-Dβ cell line, which represents self-renewing progenitor cells (ALDH^+^/Sca1^+^) that can reconstitute the mammary gland. Several miRNAs, including *miR-205* and *miR-22*, are highly expressed in mammary progenitor cells, while others are depleted, including *let-7* and *miR-93* [135,136]. In the postnatal mammary gland, *miR-205* is also predominantly expressed in the basal/stem cell-enriched population [137]. A comparison of stem cell (CD24-CD44+) and non-stem cell (non-CD24-CD44+) populations isolated from primary human mammalian epithelial cells and the normal mammary epithelial cell line MCF12A allowed to identify *miR-183* and *miR-200c* as the most downregulated miRNAs in the stem cell population compared to the non-stem cell population [138].

#### 7.1.2. Roles of miRNAs in Mammary Gland Development

The roles of several miRNAs in the different biological processes of the mammary gland have been studied using transgenic mouse models or mammary cell lines. In some cases, the regulatory relationship between the miRNA and its targets has also been identified. From profile expression data, the authors identified miRNAs that may play roles in maintaining the function of normal mammary cells, such as stem and progenitor cells and epithelial cells.

Concerning the maintenance of stem cell characteristics, initial studies have highlighted several important miRNAs. Within COMMA-Dβ cells, Ibarra et al. [135] have shown that the depletion of *let-7b* and *let-7c* can be used to enrich self-renewing cell populations. Ectopic overexpression of *miR-22* results in increased mammary ductal side branching, accompanied by an expansion of mammary stem cells [139]. *miR-93* also regulates the proliferation and differentiation of normal breast stem cells isolated from reduced mammoplasties [140].

The action mechanisms of important miRNAs in stem cells have been clarified. In the postnatal mammary gland, *miR-205*, predominantly expressed in the basal/stem cell enriched population, is critical for the regulation of this cell identity. In fact, its conditional deletion results in impaired stem cell self-renewal and mammary regenerative potential. *miR-205* regulates *Naked cuticle homologue 1* (*Nkd1*) and *Ppp2r4*, the gene encoding a specific Phosphotyrosyl Phosphatase Activator (PTPA, the B56 subunit) of the dimeric form of the tumour suppressor Protein Phosphatase 2A (PP2A), which inhibits the Wnt signalling pathway, and *Angiomotin* (*Amot*), which causes Yes-associated protein (Yap) cytoplasmic retention and inactivation and directly targets the △*Np63* gene, which is required for the preservation of self-renewing capacity in epithelial structures [4,137]. The △*Np63α* gene is also targeted by *miR-203* in mammary stem cells during mammary epithelium differentiation [141].

The *miR-193b* locus, which also encodes *miR-365-1* and the *miR-6365* cluster linked to the mammary transcription factor Stat5, has a role in the activity of mammary stem and progenitor cells [142].

*miR-146b,* which is significantly more highly expressed in the mammary glands of pregnant and lactating mice than in those of virgin mice, promotes the maintenance of pregnancy-derived mammary luminal alveolar progenitors, at least partially, by regulating Stat3b [143].

*miR-31* promotes mammary stem cell activity by regulating multiple signalling pathways, including the Prolactin Receptor (Prlr)/Stat5, Transforming growth factor-β (TGF-β) and Wnt/β-catenin pathways [144].

The *miR-34* family, *miR-34a* in particular, is involved in mammary epithelium homoeostasis. *miR-34a* is expressed in luminal cells and inhibits the expansion of mammary stem cells and early progenitor cells by regulating genes involved in epithelial cell plasticity and luminal-to-basal cell transfromation. *miR-34a* acts as an inhibitor of the Wnt/β-catenin signalling pathway [145].

*miR-206* impacts a network of signalling pathways and acts as a regulator of proliferation, stemness, and mammary cell differentiation in stem-like mammary cells [146]. Among the genes upregulated by *miR-206* addition, genes involved in inflammatory responses, such as type I interferon-mediated signalling, cytokine signalling and nuclear factor kappa B (NFκB) signalling, were the most represented. Eight genes induced by *miR-206* addition are specifically increased in the stem cell population. Among them, *Nucleostemin* (*Gnl3*), *Interferon-related developmental regulator 1* (*Ifrd1*), *Nuclear fragile X mental retardation-interacting protein 1* (*Nufip1*), and *PRKC apoptosis WT1 regulator* (*Pawr*) are linked to stem cell properties and/or tumorigenesis; *Tcf7lc*, *Secreted frizzed related protein* (*Sfrp1)*, and *Ski* (encoding a TGF-β antagonist) are present in the Gene Ontology function category “somatic stem cell maintenance”, and *Centromere protein F* (*Cenpf)* is a stem cell fate-specification gene.

Epithelial to mesenchymal transition (EMT) is a conserved developmental process throughout which epithelial cells lose their epithelial properties and adopt a mesenchymal phenotype. Several studies have shown the importance of the *miR-200* family in this transition in the mammary gland. Eades et al. [147] observed downregulation of *miR-200* family members between normal mammary epithelial cells and cells that had undergone EMT-like transformation. SIRT1, a key class III histone deacetylase whose upregulation is associated with EMT, is correlated with *miR-200a* downregulation. Moreover, loss of the tumour suppressor p53, which has a role in regulating both EMT and EMT-associated stem cell properties, leads to decreased expression of *miR-200c* and activated EMT, accompanied by an increased mammary stem cell population [138]. Exogenous expression of *miR-200c-141* in a mesenchymal-derivative breast epithelial cell line with stem cell properties (D492M) reversed the EMT phenotype, resulting in gain of luminal differentiation [148]. TGF-β, a secreted cytokine, regulates a variety of processes in development, including EMT.

*miR-99a* and *miR-99b* have been identified as two novel effectors of the TGF-β pathway during EMT in mammary cells [149]. DeCastro et al. [120] showed that *miR-203*, by targeting △*Np63α,* is able to disrupt activities associated with mammary stem cells but also to promote mesenchymal to epithelial transition. EMT is induced by overexpression of *miR-221*, which is more highly expressed in stem-like and myoepithelial cells than in luminal cells.

*miR-221* acts in EMT by targeting *Ataxin-1* (*ATXN1*), a polyglutamine protein that alters cell morphology by interacting with microtubules during neuronal development and activating E-cadherin expression [150]. Increased *miR-221* in mammary stem cells promotes myoepithelial differentiation, whereas its downregulation promotes luminal differentiation.

Some authors have focused their studies on the impact of miRNAs on mammary gland formation at different stages. The crucial role of miRNAs in the initiation of mammary gland formation has been demonstrated by Lee et al. [151]. In fact, *miR-206* is involved in mammary bud development during embryonic development by regulating the Wnt pathway, which is essential for mammary gland development [151].

Ucar et al. [152] have shown that the *miR-212/132* family is indispensable during mouse mammary gland development, particularly for the outgrowth of ducts. It is interesting to note that mammary transplantation experiments showed that *miR-212/132* family is necessary in the stroma but not in the epithelia. This could be explained by their exclusive expression in the stroma. In 3D culture of a breast epithelial cell line with stem cell properties, Hilmarsdottir et al. [148] demonstrated that *miR-200c-141* is involved in the formation of branching epithelial structures. Co-expression of *miR-200c-141* and Δ*Np63* in the D492M cell line restores branching morphogenesis, underlining the requirement for both luminal and myoepithelial elements. The mammary tissue of virgin (8-week-old) mice lacking *miR-21* has fewer secondary ductal branching than that of wild-type mice [153]. Studies on lumen formation have shown the role of miRNAs. *miR-142-3p* knockdown affects the structure and function of the mammary gland, resulting in a higher milk-producing capability due to an increased number of lobules and ducts [131].

Comparative analysis of miRNA expression in MCF7 cells *versus* MCF7/CEACAM1 cells (where CEACAM1 induces lumen formation) revealed that two miRNAs were significantly downregulated (*miR-30a-3p* and *miR-342–5p*) [154]. The regulation of lumen formation by *miR-342* involves at least two of its known targets, *DNA-binding protein inhibitor ID4* and *DNMT1*. Le Guillou et al. [129] showed that the overexpression of *miR-30b-5p* in the mouse mammary gland provokes a reduction in the size of the alveolar lumen during lactation. The involution stage is also perturbed by the misregulation of miRNAs. The overexpression of *miR-30b-5p* in the mouse mammary gland during involution results in the persistence of mammary epithelial differentiated structures, suggesting a delay in the involution process. Llobet-Navas et al. [97] showed that the *miR-424(322)/503* cluster is an important regulator of epithelial involution. The regression of secretory acini is compromised in the absence of *miR-424(322)/503*, which regulates cell survival and death decisions by targeting *B-cell lymphoma 2* (*BCl2*) and *Insulin growth factor 1 Receptor* (*Igf1R*).

The major functions of mammary epithelial cells based on their proliferation ability or differentiation status are regulated by miRNA expression (Figure 4).

Transfection of *miR-221*, which is highly expressed at peak compared with early lactation [101], into cultured bovine mammary gland epithelial cells inhibits cell proliferation and reduces the viability of these cells [155]. Dual luciferase assays have revealed that *Stat5a*, *Stat3*, and *Insulin receptor substrate 1* (*Irs1*), key genes in the PI3KAkt/mTOR and JAK-STAT signalling pathways, bind directly to *miR-221*. In a cell culture experiment, Cui et al. [128] showed that *miR-126-3p*, which is among 15 miRNAs that are highly expressed in the mammary glands of several species, inhibits the expression of progesterone receptors as well as the proliferation of mammary epithelial cells. *miR-24-3p,* which is abundantly expressed in mammary tissue, enhances proliferation. Through luciferase assays in immortalized bovine mammary epithelial cells (MAC-T), *miR-24-3p* has been shown to target the 3′UTR of *Multiple endocrine neoplasia type 1* (*Men1*) [156]. *miR-101a* is also able to regulate cell proliferation by altering *Cyclooxygenase 2* (*Cox2*) expression [127]. *miR-142-3p* knockdown in mouse mammary epithelial cells increases proliferation but not viability, induces cell cycle progression, decreases apoptosis, and increases the expression of triglycerides and β-casein, which are markers of differentiated mammary epithelial cells. Moreover, *miR-142-3p* acts by regulating multiple PrlR-mediated signalling pathways [131]. *miR-143*, which influences the apoptosis of goat mammary epithelial cells cultured in vitro, targets *Nedd4 family-interacting protein 1* (*Ndfip1*) [157]. Liao et al. [158] showed that *miR-214*, by regulating lactoferrin, is directly involved in mammary epithelial cell apoptosis regulation. Using bovine mammary epithelial cell transfection experiments, Li et al. [159] demonstrated *miR-15* involvement in the viability of mammary epithelial cells, and Cui et al. [130] showed that *miR-139* promotes proliferation by targeting the *GH Receptor* (*GHR*). *miR-21-3p*, whose function is suppressed by the transcription factor STAT3, which downregulates its transcription, promotes the proliferation of bovine mammary epithelial cells by targeting the *Insulin-like growth factor-binding protein 5 (Igfbp5)* gene [160]. Bioinformatics analysis suggested that *miR-3031* and *Igfbp5* are key signalling factors that regulate cell proliferation and protein synthesis in goat mammary epithelial cells. Chen et al. [161], using *miR-3031* mimics, showed that *miR-3031* activated the PI3K-AKT-mTOR pathway and increased *β-casein* expression by downregulating *Igfbp5*. In bovine mammary epithelium, Li et al. [162] showed that the function of *miR-486* is indispensable for regulating *Phosphatase and tensin homologue deleted on chromosome ten* (*Pten*), which reduces the differentiation of mammary epithelial cells [163]. Yoo’s study [142], which identified the *miR-193b* locus as a *Stat5* target in mammary epithelium, revealed its role in stem cell activities but also in controlling mammary epithelial differentiation. Heinz et al. [99] have shown that *miR-150* decreases between late pregnancy and lactation and is crucial for lactation. In fact, pups nursed by transgenic mice constitutively expressing *miR-150* exhibit a dramatic decrease in survival. These data support the hypothesis that a decrease in miRNAs in late pregnancy serves to allow translation of targets crucial for lactation.

Hormones play important roles in the control of mammary gland biology, and miRNA expression is under hormonal control. In bovine mammary epithelial cells, Muroya et al. [164] have shown that the production of milk-related miRNAs is influenced by the lactogenic hormones insulin, prolactin, and glucocorticoids. In goat mammary epithelial cells incubated with PRL, DNMT1 expression is increased, which leads to DNA methylation of the CpG island upstream of *miR-135b,* thereby inhibiting its transcription [165]. To identify synergistic miRNAs, Lin et al. [166] screened miRNAs differentially expressed during the lactation which respond positively to prolactin. Correlation analyses among the expression levels of four miRNAs (*miR-23a, miR-27b, miR-103* and *miR-200a*) and experiments involving their overexpression in goat mammary epithelial cells allowed to identify miRNAs that synergistically regulate milk fat synthesis in dairy goats.

Some miRNAs also contribute to lactogenic hormone induction of cellular differentiation; one such miRNA is *miR-200a* [167]. *miR-15a* and *miR-139* are involved in the regulation of *GHR* gene expression; consequently, they are important for mammary gland development, lactation, and milk composition, functions controlled by a complex interplay of endocrine hormones acting together, in particular GH, estrogen, progesterone, and PRL [130,159]. *miR-135a* is a direct regulator of *PrlR*, a specific receptor important for physiological functions in regulating mammogenesis and lactogenesis [168].

#### 7.1.3. Roles of miRNAs in Mammary Gland during Lactation

In regard to studies concerning the role of mammary miRNAs on milk composition, few articles describe the impact of miRNAs on milk protein synthesis.

To highlight miRNAs involved in milk component synthesis in dairy cow mammary epithelial cells, Bian et al. [169] showed that the inhibition of *miR-29s* causes global DNA hypermethylation and increases the methylation levels of the promoters of important lactation-related genes, including *Csn1S1*, *Elf5*, *Peroxisome proliferative activated receptor γ* (*Pparγ), Sterol regulatory element binding transcription protein 1* (*Srebp1*), and *Glucose transporter type 1* (*Glut1*). Moreover, the overexpression of *miR-152* leads to a strong decrease in DNMT1 expression, as well as a reduction in the global rate of 5-meC in mammary epithelial cells [170]. Studies describing miRNAs involved in the expression of milk protein genes, such as *casein* (*miR-3031* [161], *miR-15* [159], *miR-139* [130], *miR-101a* [127], *miR-142-3p* [131]), have highlighted the aforementioned genes as markers of the differentiation status of mammary epithelial cells (see before) rather than with the goal of understanding the role of miRNAs in their regulation. Indirectly, by showing that *miR-24-3p* regulates genes involved in the PI3K/Akt/mTOR and JAK/Stat5 pathways, which regulate milk protein synthesis, Qiaoqiao et al. [156] highlighted the importance of this miRNA in this process. miRNAs are also involved in milk calcium concentration. In fact, *miR-99-3p* was able to increase the intracellular calcium level by decreasing *ATPase plasma membrane Ca2+ transporting 1* (*Atp2B1*) in goat mammary epithelial cells [171].

**Figure 6:**
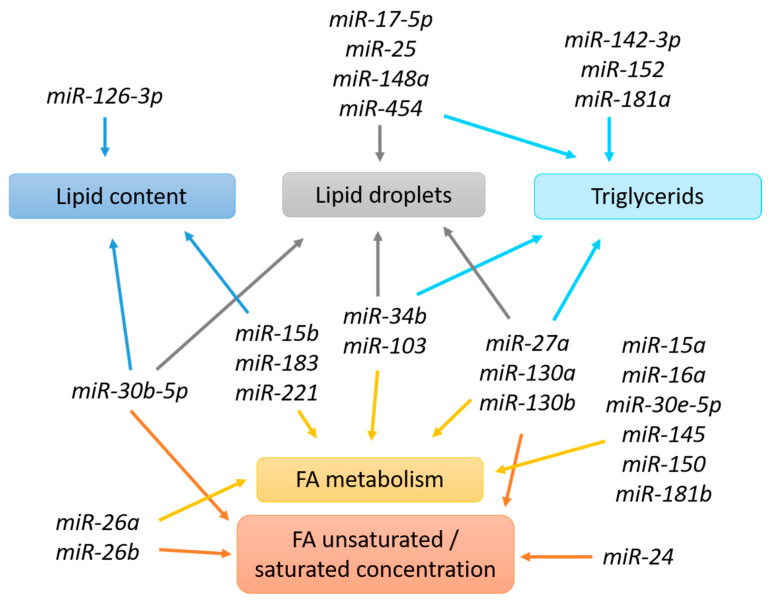
miRNAs involved in the regulation of lipid biosynthesis in mammary epithelial cells. Colour-coded arrows show which processes the mentioned miRNAs are involved in.

In contrast, several studies have characterized miRNAs involved in the regulation of milk fat biosynthesis (Figure 6). Among them, two studies have used transgenic mouse models, and the effects of this misregulation on miRNAs allowed to identify miRNAs important for lipid biosynthesis. Le Guillou et al. [129] showed that the overexpression of *miR-30b-5p* in the mouse mammary gland provokes lipid droplet formation and secretion failures and modifies milk fatty acid composition. Its overexpression provokes a downregulation of *Atl2* (a member of the ATLASTIN GTPase group described as playing a key role in lipid droplet formation) expression and changes to endoplasmic reticulum morphology [172]. Heinz et al. [99] observed a defect in lactation in transgenic dams constitutively expressing *miR-150*. In fact, the protein products of the predicted *miR-150* targets *Fatty acid synthetase (Fasn)*, *Oleoyl-ACP Hydrolase* (*Olah*), *Acetyl-CoA carboxylase α* (*Acaca*), and *Stat5b* were significantly decreased, and lipid profiling revealed a significant reduction in fatty acids synthesized by the *de novo* pathway in mammary epithelial cells of transgenic mice.

Several teams have used mammary epithelial cell culture models (goat, bovine, buffalo and human mammary epithelial cells) to study the roles of specific miRNAs in lipid metabolism. Several miRNAs studied (*miR-15b, miR-24, miR-26 family, miR-27a, miR-103, miR-126, miR-130* and *miR-145*) were chosen on the basis of their previously characterized roles in adipocytes.

The expression of *miR-15b*, which is regulated by the steroid hormones estradiol and progesterone, is lower during lactation and negatively correlated with lipid synthesis proteins, suggesting that it may be involved in lipid synthesis and milk production [173]. Additional experiments have shown that the inhibition of *miR-15b* expression increases the lipid content in mammary epithelial cells through an increase in the level of the lipid synthesis enzyme fatty acid synthase. The overexpression or downregulation of *miR-24* in goat mammary epithelial cells strongly affects fatty acid profiles, particularly unsaturated fatty acid concentrations. *miR-24* also causes changes in triacylglycerol content and the expression of *Fasn*, *Srebp1*, *Stearoyl-CoA Desaturase* (*Scd*), *glycerol-3-phosphate acyltransferase mitochondrial* (*Gpat*), and *acetyl-CoA carboxylase 1* (*Acaca*). Luciferase reporter assays confirmed that *Fasn* is a direct target of *miR-24* [174]. Overexpression of *miR-27a* downregulates triglyceride accumulation and decreases the ratio of unsaturated/saturated fatty acids and lipid droplet formation in mammary epithelial cells by affecting the expression of mRNAs related to milk fat metabolism, such as *Pparγ* [175,176]. The genomic loci of *miR-26a* and *miR-26b* have been localized to the introns of genes in the *C-terminal domain RNA polymerase II polypeptide A small phosphatase* (*Ctdsp*) family. The downregulation of *miR-26a/b* and their host genes in goat mammary epithelial cells decreased the expression of genes related to fatty acid synthesis (*Pparγ*, *liver X receptor α* (Lxrα or *Nr1H3*), *sterol regulatory element-binding transcription factor 1* (*Srebf1)*, *Fasn*, *Acaca*, *Glycerol-3-phosphate acyltransferase* (*Gpam*), *Lipin 1* (*Lpin1)*, *Diacylglycerol acyltransferase 1* (*Dgat1*) and *Stearyl-coenzyme A desaturase 1* (*Scd1*)), triacylglycerol accumulation and unsaturated fatty acid synthesis. Luciferase reporter assays confirmed *Insulin-induced gene 1* (*Insig1*) as a direct target of *miR-26a/b* [177]. Studies on the relationship of the *miR-26* family and its host genes with milk composition revealed that their expression are associated with total fat yield and fatty acid content but not milk protein or lactose content. Moreover, a significant positive correlation was detected for this miRNA family and the C16:1 and C18:3 fatty acid contents [178]. The overexpression of *miR-103* in mammary epithelial cells increases the transcription of genes associated with milk fat synthesis, resulting in an upregulation of fat droplet formation, triglyceride accumulation and the proportion of unsaturated fatty acids [104,179]. *miR-126-3p,* which is differentially expressed at various stages of murine mammary gland development, exhibits a negative correlation with *Fasn* expression. Its overexpression in MFC-10A cells decreases lipid content with a reduction in *Fasn* and *Insig1* expression [180]. The overexpression of *miR-130a* significantly decreases cellular triacylglycerol levels and suppresses lipid droplet formation in bovine mammary epithelial cells [181]. *miR-130a* also significantly affects the expression of mRNAs related to milk fat metabolism, such as *Pparγ, Fatty acid binding protein 3* (*Fabp3)*, *Perilipin 2* (*Plin2)*, *Fatty acid transport protein 1* (*Fatp1)*, *CCAAT enhancer binding protein β* (*C/EBPβ)*, *CCAAT enhancer binding protein α* (*C/EBPα*). Among these, *Pparγ* is a direct target of *miR-130a*. Moreover, in goat mammary epithelial cells, Chen et al. [182] showed that overexpression of *miR-130b* potently impairs adipogenesis by repressing the expression of *Pparγ coactivator-1α* (*Ppargc1a*), a major regulator of fat metabolism. The down-regulation of *miR-145* inhibits triacylglycerol and cholesterol contents by regulating the expression of fatty acid metabolism-related genes in goat mammary epithelial cells by targeting *Insig1* [183,184].

Some authors have focused on the identification of the mechanism of action or targets of miRNAs according to variations in expression observed in the mammary gland either during different reproductive cycle stages or with different milk production status.

In mice, *miR-142-3p* is differentially expressed in virgin, pregnancy, lactation, and involution stages. Its knockdown in mouse mammary epithelial cells increased proliferation but not viability and increased the expression of triglycerides by the regulation of multiple PrlR-mediated signalling pathways [131]. In goats, the analysis of the correlation between differentially expressed miRNAs in mammary tissue and the fatty acid composition of milk allowed to determine that the *miR-183* level is highly and positively correlated with the fatty acid content in milk and that *miR-183* inhibits milk fat metabolism by targeting *Mammalian Ste20-like kinase 1 (Mst1)* [185]. Recently, in research undertaken to better understand the internal relationship between milk quality and lipid metabolism in cows, Jiao et al. [186] showed that *miR-183* contains a CpG island in its promoter region and that Prl inhibits its expression by methylation of this region. The downregulation of miR-183 in turn leads to the upregulation of the expression of the target gene *Irs1*, which ultimately leads to changes in fatty acid metabolism. *miR-16a,* which is one of the miRNAs that is differentially expressed in the mammary gland during lactation, regulates biological processes associated with intracellular triacylglycerol, cholesterol and unsaturated fatty acid synthesis through *Large Tumour Suppressor 1* (*Lats1*) [109]. *miR-221,* identified in milk and adipocytes, is more highly expressed in stem-like and myoepithelial cells than in luminal cells in mammary tissue [150]. *miR-221*, the expression of which is regulated by steroid hormones estradiol and progesterone, can also regulate lipid metabolism in mammary epithelial cells through modulation of the expression of genes related to lipid synthesis, such as *Fasn*, *Acyl-CoA synthetase long chain family member 1* (*Acsl1*), *Elf5*, *Insig1*, *Pparγ* and *Nr1H3*. Moreover, the milk proteins α-casein and β-casein and the glucose transporter GLUT1 are similarly regulated by *miR-221*, suggesting that this miRNA regulates milk lipid formation and play a role in glucose transportation and milk protein synthesis [187]. *miR-148a and miR-17–5p*, such as *Ppargc1a* (a major regulator of fat metabolism) and *Pparα* (an important regulator of fatty acid oxidation), are highly expressed in the goat mammary gland during the early lactation and non-lactating periods. *miR-148a* cooperates with *miR-17–5p* to regulate triacylglycerol synthesis and milk fat droplet accumulation by targeting *Ppargc1a* and *Pparα*, respectively, in goat mammary epithelial cells [188]. *miR-152*, whose expression is increased significantly in mammary epithelial cells of cows with high milk production [170,189], could influence triglyceride production and suppress apoptosis via the expression of its target genes *Acetyl-coenzyme A acyltransferase 2* (*Acaa2*) and *Hydroxysteroid 17-β dehydrogenase 12* (*Hsd17B12*) [190] and *Membrane uncoupling protein 3* (*Ucp3*) [191]. The overexpression of *miR-25*, which has an inverse relationship with milk production, significantly represses triacylglycerol synthesis and lipid droplet accumulation, and expression of its mimic in goat mammary epithelial cells reduce the expression of genes involved in lipid metabolism (*Srebp1*, *Fasn*, *Pparγ*, *Gpam*). *Peroxisome proliferative activated receptor γ coactivator 1 β* (*PGC-1beta*) has been identified as a direct target of *miR-25* [114]. *miR-34b* mimic transfection in bovine mammary epithelial cells reduces the content of intracellular triacylglycerol and lipid droplet accumulation; moreover, overexpression of miR-34b inhibits the mRNA expression of lipid metabolism-related genes such as *Pparγ*, *Fasn*, *Fatty acid binding protein 4* (*Fabp4*), and *C/EBPα*. Furthermore, mRNA *Decapping enzyme 1A* (*Dcp1A*) is a direct target of *miR-34b,* revealing a novel *miR-34b*–*Dcp1A* axis that has a significant role in regulating milk fat synthesis [192]. Fat droplet accumulation and triglyceride production are inversely correlated with *miR-454* expression. This miRNA is able to target *Pparγ* 3′UTR [193]. *miR-181a* expression increases between the dry and early lactation periods [101] and is able to regulate the expression of *Acsl1*, which plays a role in activating fatty acids destined for triacylglycerol synthesis, in bovine mammary epithelial cells [194]. By screening for miRNAs expressed in the goat mammary gland during peak- and late-lactation periods, Chen et al. [195] found that *miR-181b* is differentially expressed. Its overexpression impairs fat metabolism, while its knockdown promotes fat metabolism. They have also shown that miR-181b regulates the Hippo pathway by directly regulating *Irs2, Lats1* and *Yes-associated protein 1* (*Yap1)* genes.

*miR-30e-5p* and *miR-15a,* which are differentially expressed in the mammary gland between peak lactation and dry periods, synergistically regulate fatty acid metabolism in goat mammary epithelial cells via *Lipoprotein receptor 6* (*Lrp6),* a component of cell-surface receptors for Wnt proteins, and *Yap1,* playing a role in promoting cell growth and inhibiting apoptosis [196].

These miRNAs might be useful targets for influencing lipid production and milk yield.

Mammary gland development and dairy potential differ among breeds. Farm animals are historically selected for specific traits, such as dairy production. These animals are therefore good models in which to study the impact of genetic background on miRNA variations.

Mammary miRNA expression profiles have been characterized in swine breeds with divergent phenotypes [126], dairy and beef breed heifers [119] and two breeds of dairy cows (Normande and Holstein) [121]. In these three studies, differentially expressed miRNAs between breeds were identified. Among differentially expressed miRNAs, Peng et al. [126] found breed-specific miRNAs. These studies suggest a potential role for miRNAs in mammary tissue plasticity and milk component synthesis, both of which are able to change to milk production [121,126], as well as mammary stem cell activity [119].

The connection between miRNA expression, milk yield, and component traits has also been studied in cows. Correlations were observed between modules of miRNAs (8 modules with 32 to 164 miRNAs each) and milk yield, lactose, and somatic cell count but not fat %, protein %, or milk urea nitrogen [118]. Wang et al. [102] studied 15 Holstein cows with similar genetic backgrounds producing milk with different compositions (high-protein/high-fat or low-protein/low-fat milk). Thirty-eight miRNAs were differentially expressed between the two groups. These miRNAs putatively negatively regulate 253 differentially expressed mRNAs, which are enriched in lipid biosynthesis processes and amino acid transmembrane transporter activity. Their results suggest that differentially expressed miRNAs might play roles in milk quality regulation.

Shen et al. [189] performed an original study by using primary mammary epithelial cells derived from two Chinese Holstein dairy cows with extreme differences in milk fat percentage. They compared the two miRNomes of these cells and showed that 97 miRNAs were differentially expressed between the two samples. Among them, three miRNAs (*bta-miR-33a*, *bta-miR-152*, *bta-miR-224*) have, as predicted, target genes related to the lipid metabolism pathway. Triglyceride production decreased, and the apoptosis rate increased, after overexpression of *miR-224* in mammary epithelial cells, which probably regulates the expression of *Acyl-CoA dehydrogenase* (*Acadm*) and *Aldehyde dehydrogenase 2* (*Adlh2*) [197]. *miR-152* affects the intracellular triacylglycerol content by targeting *Ucp3* [191].

### 7.2. lncRNAs

#### 7.2.1. Characterization of lncRNA in Mammary Gland

In whole-genome tiling arrays, Perez et al. [198] identified a new group of abundantly expressed lncRNAs and found that a subset of them are highly evolutionarily conserved. Then, they characterized 15 of them in different human tissues, such as the mammary gland, by quantitative RT-PCR. In humans, these descriptions by large-scale analyses have been completed by the SAGE-seq study performed by Maruyama et al. [199].

The panoramic view of lncRNAs in the bovine mammary gland has allowed the identification of 184 intergenic lncRNAs (lincRNAs) [200]. Many of them are located in quantitative trait loci (QTLs). In particular, 36 lincRNAs were found in 172 milk-related QTLs. Further analyses indicate the involvement of lincRNAs in several biological functions and different pathways. Such extensive annotation of the mammary gland and associated lincRNAs helps further our understanding of bovine mammary gland biology [200].

To identify lncRNAs involved in mammary gland development, Askarian-Amiri et al. [201] performed microarray analyses using mouse mammary gland RNA from different stages of its development (day 15 pregnant, day 7 lactation, and day 2 involution). In this study, almost 100 lncRNAs with differential expression between the different stages were identified. As differentially expressed coding genes showing enrichment in genes involved in regulation of cell growth and size and with response to hormone stimulus, the authors anticipated that the differentially expressed lncRNAs should be similarly relevant to the biological processes underlying mammary development.

LncRNAs act as competing endogenous RNAs (ceRNAs) to regulate gene expression. The interactions of ceRNAs have potential roles in gene expression and cell phenotypes. Studies have synthesized various expression profile data to construct a network of the lncRNAs, mRNAs and miRNAs involved in mammary gland biology.

The expression profiles of lncRNAs and mRNAs from the Chinese Holstein mammary gland in the dry and lactation periods have been studied [202]. In total, 3746 differentially expressed lncRNAs and 2890 differentially expressed genes were identified. Functional enrichment analysis of target genes of lncRNAs indicates that these genes are involved in lactation-related signalling pathways, including the cell cycle, JAK-STAT, cell adhesion, and PI3K-Akt signalling pathways.

Yu et al. [203] sequenced mRNA, miRNA and lncRNA in goat mammary tissue at two periods in lactation (early and mature). Their data show that the ceRNAs (lncRNA-mRNA) upregulated during the mature lactation stageare associated with milk content synthesis and their metabolism. These data correlate with the function of this stage - a period in which a continuous production of large amounts of milk that is rich in proteins, lipids, amino acids and other nutrients is observed. In contrast, the ceRNAs upregulated during early lactation are associated with PI3K-AKT pathways and ECM-receptor interactions; this fulfils the functional role of preparing the mammary gland for full lactation. Together, these results suggest that ceRNAs have synergistically roles during different developmental periods to regulate functions which control lactation control.

The RNA expression profiles at peak and late lactation have been characterized using RNA sequencing technology in cow mammary glands [204]. Functional lncRNA-mRNA coexpression pairs were constructed to infer the function of lncRNAs. More than 1000 putative lncRNAs were identified, 117 of which were differentially expressed between peak and late lactation stages. Seventy-two differentially expressed lncRNAs were coexpressed, along with 340 different protein-coding genes. The KEGG pathway analysis shows that target mRNAs for differentially expressed lncRNAs are mainly related to lipid and glucose metabolism, including the PPAR and 5′adenosine monophosphate-activated protein kinase signalling pathways. Further bioinformatics and integrated analyses revealed that 12 differentially expressed lncRNAs (*XLOC_000752*, *XLOC_306924*, *XLOC_274111*, *XLOC_517858*, *XLOC_518578*, *XLOC_555176*, *XLOC_626085*, *XLOC_000752*, *XLOC_306924*, *XLOC_274111*, *XLOC_518578*, and *XLOC_626085*) may play important roles in bovine lactation [204].

Recently, Ji et al. [205] profiled lncRNA expression in the mammary gland tissue of Laoshan dairy goats from three different lactation periods (early, peak and late lactation). A total of 39,863 transcripts were detected, including 37,249 coding mRNAs and 2614 lncRNAs. Among these lncRNAs, 21 lncRNAs (six known and 15 novel lncRNAs) were identified as precursors for 461 known miRNAs. In total, 2381 lncRNAs are expressed in at least one of the three mammary gland lactation periods. They are found 573 differentially expressed lncRNAs and 1237 differentially expressed mRNAs in mammary gland development. The functions of lncRNAs and the corresponding genes have been predicted: 489 genes are annotated with biological processes, specifically, cellular processes, single-organism processes, biological regulation, metabolic processes, response to stimuli, developmental processes, multicellular organismal processes, organization or biogenesis, and localization.

The differentiation of the breast induced by the hormones of pregnancy plays a major role in breast cancer protection, the identification of differentiation-associated molecular changes, which persist in the breast until menopause, has been performed [206]. Transcriptome analyses of the breasts of 42 nulliparous and 71 parous postmenopausal women revealed upregulated genes controlling chromatin organization, transcription regulation, splicing machinery, and mRNA processing, as well as lncRNAs such as *XIST*, *NETA1*, *MALAT-1*, *CXorf50B*, *NCRNA00173* and *NCRNA00201*. These lncRNAs are known to recruit polycomb proteins that lead to the condensation of chromatin.

In 2018, Cai et al. [207] performed the first study on global expression profiling of lncRNAs and mRNAs related to milk protein traits by studying mammary tissue samples from Holstein cows with extremely high or low milk protein percentage phenotypes. They identified 6450 lncRNAs, among which 31 lncRNAs were identified to be differentially expressed, and 8 and 10 lncRNAs were expressed in only the high milk protein or in only the low milk protein groups, respectively. To better understand the relationship between lncRNAs and milk protein traits, they selectively analysed the 2868 lncRNA-mRNA pairs in which both lncRNAs and their neighbouring or expression correlated genes are differentially expressed between high milk protein and low milk protein groups. According to the integrated study, 30 lncRNAs potentially regulate 34 genes that are involved in milk protein synthesis. For example, they proposed that *XLOC_059976* acts as a regulatory molecule by enhancing the expression of *ciliary neurotrophic factor receptor* (*Cntfr*) and affecting the secretion of milk proteins.

These results provide a resource for lncRNA research in the mammary gland, with important information and insights into the synthesis of milk proteins, as well as potential targets for the future improvement of milk quality.

#### 7.2.2. Roles of lncRNA in Mammary Gland

The role of lncRNAs in mammary gland biology remains largely unexplored. In comparison with proteins and miRNAs, lncRNAs are relatively poorly annotated and characterized. However, a few lncRNAs have been documented in the literature as having potential roles in mammary gland development (Figure 4); these are presented below.

Standaert’s [208] study unequivocally identified the first physiological function of one of the most abundant lncRNAs, *Nuclear Paraspeckle Assembly Transcript 1* (*Neat1*), in mammary gland development and lactation. The ablation of *Neat1* results in abnormal mammary gland morphogenesis as well as additional defects in lactation. This phenotype is caused by the decreased ability of cells to sustain high rates of proliferation during lobulo-alveolar development. *Neat1* is required for mammary gland branching morphogenesis, lobular-alveolar development, and lactation.

*HOX transcript antisense intergenic RNA* (*HOTAIR*) is localized within the HoxC gene cluster and is regulated by estrogen. *HOTAIR* downregulates *HoxD* gene expression, which is necessary for the mammary epithelium ductal system differentiation during pregnancy [209]. PRC2 recruitment to the genomic regions of target genes, repressing gene transcription is increased by *HOTAIR* [210]. eEven though the PRC2 complex has been predicted to maintain differentiated alveolar cells in the involuted gland, knockout of *HOTAIR* does not showed phenotype modification. The role of *HOTAIR* remain controversial, and its specific function remains to determined.

*Epithelial cell Program Regulator* (*EPR*), an intergenic lncRNA expressed in epithelial tissues, is downregulated by TGF-β.Its expression largely changes the transcriptome, by increasing the acquisition of epithelial traits, and by reducing cell proliferation. These data were observed in mammary cells culture, and after murine transplantation. *EPR* produces a small peptide localized to epithelial cell junctions, but the RNA molecule provokesthe vast majority of *EPR*-induced gene expression changes. *EPR* interacts with chromatin and modifies *Cdkn1a* gene expression by affecting its transcription and mRNA decay. *EPR* enables to control proliferation of epithelial cells by modulating waves of gene expression in response to TGF-β [211].

*Steroid Receptor RNA activator 1* (*SRA1*) is a lncRNA that acts as a nuclear coactivator of steroid hormone receptors [212,213] as well as non-steroid receptors and transcription factors. To assess its function in vivo, a transgenic mouse model was generated to enable robust human *SRA* expression. No change is observed during early development, but in virgin transgenic mice, elevated proliferation and apoptosis in the mammary gland are observed. Activation of *SRA1* leads to an increase in cell proliferation and differentiation and to abnormally early development of the ductal epithelium. *SRA1* also regulates cell death, as epithelial hyperplasia is found to be accompanied by increased apoptosis [214]. A relationship between the roles of *SRA1* and steroid hormone receptors is underlined in mammary gland development, as well as a role in the maintain of healthy breast function by regulating apoptosis.

*ZNFX1 antisense RNA 1* (*Zfas1*), an antisense lncRNA that overlaps with the promoter region of the gene *ZNFX1*, is one of the most highly and differentially expressed lncRNAs during mammary gland mouse development [201] and is localized to the ducts and alveoli of the mammary gland. Its knockdown in a mouse mammary epithelial cell line highlights its dual role in cellular proliferation and differentiation.

*Pregnancy-induced noncoding RNA* (*PINC*) is differentially expressed in the mammary gland; it is upregulated in alveolar cells during pregnancy and downregulated during the transition from late pregnancy to early lactation, periods of terminal differentiation of epithelial cell to cells which milk product, and it is again elevated during involution. *PINC* regulates cell cycle progression [215] and inhibits differentiation [216]. It interacts with a component of PRC2, Retinoblastoma associated protein 46 (RbAp46), suggesting that *PINC* may affect its role in differentiation through modification of chromatin [216].

The imprinted lncRNA *H19* is induced by estrogen in the mouse mammary gland. Its expression decreases during prepubertal development, increases during both puberty and pregnancy, decreases during lactation and then increases once again during involution, indicating that it is not necessary for terminal differentiation but may instead function in proliferation, migration, and preterminal differentiation [217]. It is expressed in TEBs during puberty and in alveolar cells during pregnancy, and its expression is controlled by steroid hormones [218]. Knocking down the *H19* gene in ERα-positive human luminal progenitors decreased their colony-forming potential, a phenotype that could not be rescued by the addition of estrogen, showing that the estrogen–ERα–*H19* signalling axis is involved in the regulation of the proliferation and differentiation potentials of luminal progenitors [219].

### 7.3. miRNA-lncRNA Interactions

miRNAs are regulated transcriptionally and post-transcriptionally by a class of lncRNAs known as ceRNAs. They act as sponges or decoys to titrate miRNAs away from their target mRNAs and inhibe their activity [220]. Paci et al. [221] performed computational analyses to assess whether specific lncRNAs function as miRNA decoys in the breast epithelium. They built networks of miRNA-mediated sponge interactions by multivariate analysis. Complex regulatory networks of miRNA-mediated interactions were found to bridge target mRNAs and lncRNAs. In the network, the authors found clear separation into two internally well-connected components: a larger component (1354 nodes and 32,375 edges) mainly dominated by *miR-200* family members and a smaller component (378 nodes and 954 edges) mainly controlled by *miR-452*. In terms of functional annotation, the larger sub-network is enriched in cell-cell adhesion, whereas the smaller sub-network is enriched in cellular metabolic processes. These authors therefore proposed the components to represent pure sponge and mixed TF-sponge modules. The first module employs PTENP1, a growth-suppressive lncRNA that appears to regulate the expression of a member of the HRAS-like suppressor family (HRASLS5), by antagonizing *miR-135b*. The second engages PVT1 as a competitor of CDH1 for binding to the *miR-200* family and ZEB1 as both a transcriptional repressor of CDH1 and a target of the *miR-200* family.

Yang et al. [202], by studying the expression profiles of lncRNAs and miRNAs from the Chinese Holstein mammary gland in the dry and lactation periods, identified the interaction between lncRNAs and their potential miRNAs. They found that *miR-221* might interact with the lncRNAs *TCONS_00040268*, *TCONS_00137654*, *TCONS_00071659* and *TCONS_00000352*, revealing that these lncRNAs might be important regulators of the lactation.

Cai et al. [207] performed a study on the profil of expressed lncRNAs related to milk protein composition by studying mammary samples from Holstein cows with extremely high or low milk protein percentage phenotypes. They identified a total of 4972 lncRNA transcripts predicted to be targeted by 788 bovine miRNAs. Among them, 206 lncRNAs were targeted by *miR-15a, miR-486, miR-135, miR-101a, miR-152* and *miR-139*, which are reportedly involved in milk protein synthesis. One differentially expressed lncRNA, *XLOC_059976*, was predicted to be targeted by *miR-139* and *miR-152*, which implies that *XLOC_059976* could be a regulator for the milk protein synthesis.

The lncRNA *NONBTAT017009.2* was found to interact with *miR-21-3p* and function as a ceRNA to upregulate the expression of *Igfbp5* while inhibiting the expression of *miR-21-3p* [160].

### 7.4. Circular RNA

RNA-seq has been used to characterize the circRNAs in the human mammary gland [222], at two different lactation stages in rats [223] and bovines [224], and in two breeds of sheep with different milk production characteristics [125].

Xu et al. [222] found that the number of circRNAs in human mammary gland tissue is higher than that in other adult tissues, demonstrating the tissue-specific expression of circRNAs.

In rat mammary glands at two different lactation stages, 6824 and 4523 circRNAs were identified [223]. Only 1314 circRNAs are expressed at the two stages and numerous circRNAs are specifically expressed at different lactation stages. The majority of the candidate circRNAs map to intergenic regions and noncoding intronic. An enrichment of protein kinases and related proteins among the set of genes encoding circRNAs were revealed by DAVID analysis. Interestingly, four protein-coding genes (*Rev3l*, *IGSF11*, *MAML2*, and *LPP*) that also transcribe high levels of circRNAs have been reported to be involved in cancer.

In the cow mammary gland on day 90 and day 250 postpartum, 4804 and 4048 circRNAs were identified, respectively. Among them 2231 circRNAs were co-expressed at both stages, suggesting high stage specificity in the circRNAs [224]. The enrichment of some Gene Ontology terms for the circRNA genes was different between lactation stages. Among the top 10 enriched Gene Ontology terms, vesicle, endoplasmic reticulum, and mitochondrial lumen were more common on lactation day 90. In bovine mammary glands, 80 circRNAs were produced by the four casein-coding genes (*Csn1S1*, *Csn1S2*, *Csn2*, and *Csn3*) CircRNAs from *Csn1S1* were very abundant, and three of them correspond to 36% of all the circRNAs expressed in the mammary gland on lactation day 90. Three circRNAs from *Csn1S1*, one from *Csn1S2*, and one from *Csn2* were all more highly expressed on lactation day 90 than on lactation day 250. These circRNAs have several targets in the *miR-2284* family and are predicted to target *Csn1S1* and *Csn2* mRNA, suggesting their potential involvement in regulating the expression of casein genes.

By comparing the expression profiles of circRNAs in mammary glands from sheep with different milk yields and components, Hao et al. [125] found 4906 circRNAs, among which 33 were differentially expressed between the two breeds. The parental genes of differentially expressed circRNAs were mainly enriched in heterocyclic compound binding, kinase activity, adherens junctions, the TGF-β signalling pathway, and the MAPK signalling pathway.

### 7.5. miRNA-circRNA Interactions

For the 33 differentially expressed mammary circRNAs identified by comparing two sheep breeds by Hao et al. [125], 1200 pairs of circRNA-miRNA interactions were predicted by bioinformatics. Some target miRNAs of circRNAs have been previously associated with bovine mammary gland development.

Zhang et al. [225] described a circRNA-miR-gene axis. They showed that *miR-574-5p*, which is differentially expressed during the colostrum and peak lactation stages, induces the downregulation of *Ecotropic Viral Integration site 5-like* (*EVI5L*) expression, while *circRNA-006258* relieves the inhibitory effect by adsorbing *miR-574-5p*. Since EVI5L promotes cell growth, inhibits apoptosis and is involved in triacylglycerol production, the *circRNA–006258/miR-574-5p/EVI5L* axis could regulate the cell growth and milk synthesis of goat mammary epithelial cells by sponging *miR-574-5p*.

## 8. Conclusions

At present, the importance of epigenetic mechanisms on mammary gland development and milk production is clearly established, even if all epigenetic pathways are far from fully clear. An increasing understanding of the epigenetic machinery underlying mammary gland development and function is still necessary, and future studies focusing on the crosstalk between epigenetic marks, gene expression regulation, and the signalling pathways involved will open the door to understanding mammary gland biology in molecular detail.

To date, the different types of epigenetic modulations have been studied independently; however, the targets of different pathways appear to interact, thereby constituting regulatory networks. In the future, it will be important to continue to understand the roles of each type of epigenetic factor as well as the interrelationships between different epigenetic mechanisms.

Moreover, obtaining knowledge on the epigenetic level of regulatory control may lead to new insights into mammary gland function that may lead to improvements in milk production and quality.

Mammary gland development, lactation, and milk production could be negatively impacted by the environment, as well as the health status and diet of the female. The evaluation of the roles of epigenetic mechanisms in these disruptions will be possible only if epigenetic regulation in a normal environment is characterized.

Moreover, knowledge of miRNAs is particularly useful since the miRNAs present in milk are linked to miRNA expression in the mammary gland and are easily analysed. Therefore, they could be used as biomarkers of changes in animal status and of the effects of environmental modifications on females during lactation.

## Figures and Tables

**Table 1:** Summary of epigenetic modifications and enzymes that play a role in mammary gland development and function.

Groups of Epigenetic Regulators	Group Members Involved in Mammary Gland Development and Function	Epigenetic Modification	Description
DNA methyltransferases (DNMTs)	DNMT1, DNMT3A, and DNMT3B	DNA methylation	Family of enzymes that catalyse the transfer of a methyl group (CH_3_) to cytosine in order to form 5-methylcytosine (5-mC) occurs on the 5th carbon of the pyrimidine ring. Methylation is most often found at CpGs, but has been observed in other instances.
TET methylcytosine dioxygenases	TET1, TET2, and TET3	DNA methylation	Ten-eleven translocation methylcytosine dioxygenases oxidize 5-mC to 5-hydroxymethylcytosine (5-hmC), 5-formylcytosine (5-fC), and 5-carboxylcytosine (5-caC).
Polycomb-group proteins (PcG)	EZH2, Suz12, BMI1, Pygo2	Histone (H3K27) methylation, Histone (H3K56) acetylation	Family of enzymes that catalyse the transfer of a methyl group (CH_3_) or an acetyl group (CH_3_CO) to lysine (K) residues of histone proteins.
Lysine demethylases (KDMs)	JARID1B, UTX, JHDM1B, JMJD2B	Histone (H3K4, H3K36, H3K9, and H3K27) demethylation	Enzymes that catalyse the removal of methyl groups (CH_3_) from K residues of histone proteins.
Sirtuins (SIRTs)	SIRT1	Histone deacetylation	Class III of histone deacetylases (HDACs) that catalyse NAD-dependent histone lysine deacetylation.
miRNAs	too many to enumerate	mRNA degradation	Small (~22 nt) non-coding RNAs that regulate post-transcriptional gene expression through negative regulation or mRNA degradation.
lncRNAs	too many to enumerate	Chromatin remodeling	Long (≥200 nt) non-coding RNAs that regulate gene expression through different mechanisms, including chromatin remodeling.

**Table 2:** Lactating mammary gland miRNomes.

Species	Microarray Characterization	Small RNA Sequencing Characterization
Mouse	[95,96]	[98,108]
Rat		[115]
Cow	[119]	[93,100,108,120,121]
Buffalo		[104]
Goat		[103,110,112,113,116,122,123]
Sheep	[105]	[124,125]
Pig		[126]

## Abbreviations

ncRNA: non-coding RNA; DNMTs: DNA methyltransferases; lncRNA: long non-coding RNA; miRNA: microRNA; circRNA: circular RNA; PcG: polycomb-group proteins; TEB: terminal end bud; EMT: epithelial to mesenchymal transition; ceRNA: competing endogenous RNA.
